# Payload Identification and Gravity/Inertial Compensation for Six-Dimensional Force/Torque Sensor with a Fast and Robust Trajectory Design Approach

**DOI:** 10.3390/s22020439

**Published:** 2022-01-07

**Authors:** Jinjun Duan, Zhouchi Liu, Yiming Bin, Kunkun Cui, Zhendong Dai

**Affiliations:** 1 College of Mechanical and Electrical Engineering, Nanjing University of Aeronautics and Astronautics, Nanjing 210016, China; L16605223643@163.com (Z.L.); bingley@nuaa.edu.cn (Y.B.); kkcui@nuaa.edu.cn (K.C.); zddai@nuaa.edu.cn (Z.D.); 2 Key Laboratory of Measurement and Control of Complex Systems of Engineering, Southeast University, Ministry of Education, Nanjing 210096, China

**Keywords:** gravity compensation, inertial compensation, excitation trajectory, Fourier series, fast identification

## Abstract

In the robot contact operation, the robot relies on the multi-dimensional force/torque sensor installed at the end to sense the external contact force. When the effective load and speed of the robot are large, the gravity/inertial force generated by it will have a non-negligible impact on the output of the force sensor, which will seriously affect the accuracy and effect of the force control. The existing identification algorithm time is often longer, which also affects the efficiency of force control operations. In this paper, a self-developed multi-dimensional force sensor with integrated gravity/inertial force sensing function is used to directly measure the resultant force. Further, a method for the rapid identification of payload based on excitation trajectory is proposed. Firstly, both a gravity compensation algorithm and an inertial force compensation algorithm are introduced. Secondly, the optimal spatial recognition pose based on the excitation trajectory was designed, and the excitation trajectory of each joint is represented by a finite Fourier series. The least square method is used to calculate the identification parameters of the load, the gravity, and inertial force. Finally, the experiment was verified on the robot. The experimental results show that the algorithm can quickly identify the payload, and it is faster and more accurate than other algorithms.

## 1. Introduction

In recent years, most industrial/collaborative robots are used in non-contact operations, such as spraying, polishing, gluing, and handling. With the increasing demand for small-batch, flexible, and multi-variety operations, the demand for contact operations such as polishing, polishing, assembly, remote operation, and human–computer interaction has become increasingly significant. For the robot contact operations, the end-effector of the robot needs to accurately sense the contact force with the environment to ensure the smooth progress of the contact operation. There are many ways to sense the contact force between the end-effector of the robot and the environment: (1) Estimate the wrench based on the current [1]; (2) based on the joint force sensor, the end contact force is sensed through Jacobian transposition [2]; (3) the external contact force is directly sensed through the six-dimensional force sensor installed on the end-effector [3]. If the external contact force is accurately sensed through the current estimation method, it needs to be based on a dynamic model, and this method requires dynamic parameter identification and nonlinear control [4]. At present, it is difficult to achieve precise dynamic control by current estimation alone, and external force perception is not feasible. It is not realistic to add a torque sensor to the existing machine, and it is also difficult to control based on dynamics [5].

Compared with the first two methods, the advantages of the third method include: The joint torque sensor does not need to be added by changing the mechanical structure. The existing mechanism and control system do not need to be changed, the sensor is easily installed at the end of the flange. Through the control algorithm [6,7], the precise perception and application of the end force can be realized. When the robot performs operations, the loads that meet the requirements of different operations will be installed at the end of the robot, and the six-dimensional force sensor will generally be installed between the flange and the workpiece. In the low-speed motion of the robot, in order to accurately sense the force between the workpiece and the environment, it is necessary to eliminate the influence of the load’s center of gravity on the direct measurement value of the sensor. Further, when the robot is moving at a medium speed or a high speed, it is necessary to eliminate the influence of inertial on the measured value of the sensor.

At present, gravity compensation algorithms have been proposed in many works [8,9,10], which can be divided into three categories according to the application scenarios of gravity compensation. (1) Gravity compensation for the multi-dimensional force sensor installed at the base: a dynamic force compensation algorithm for zero value of the six-dimensional force sensor at the base is proposed [11], which solves the problem of the robot’s own gravity and inertia during the movement of the robot. However, this scheme is only suitable for collision detection in human–machine collaboration, and cannot effectively perceive the end force of the robot. (2) Gravity compensation for the multi-dimensional force sensor installed at the end-effector of the robot. To improve the robot’s perception of contact force, a robot compliance control method based on gravity compensation of force sensor is proposed [12]. By collecting data from force sensors in different poses of the robot, the parameter information of the robot load is calculated. Since the robot pose has not been specially selected, the recognition effect is not very good. In order to avoid the problem of improper posture selection, the gravity and center of gravity parameters at the load end were obtained by experimentally determining the form of gravity variation at different attitudes at the load end [13]. An optimization algorithm of gravity compensation based on genetic algorithm is proposed [14], the optimal solution model is established with the goal of minimizing the sum of squares of error, and the installation deviation angle of force sensor is obtained by genetic algorithm. A multi-objective optimization problem to ensure the recognition accuracy and ease of identification for the problem of robot identification pose selection is proposed [15], and the multi-objective particle swarm optimization method to obtain the optimal identification pose is used. Although the above algorithm can solve the problem of selecting the recognition attitude, it only performs gravity compensation and does not realize inertial force compensation. (3) Gravity compensation of each link: the gravity moment of each joint based on the principle of virtual displacement is derived [16], the electric drive to compensate the gravity of each link is used. In order to achieve gravity feedforward compensation, a PD control method based on a robot model is designed [17]. To solve the problem of tracking errors caused by the gravity of each joint linkage, an adaptive neural network model is designed [18].Further, a PD gravity compensation algorithm based on maximum gravity moment estimation and trigonometric function is proposed based on the spatial geometric characteristics of manipulator and the principle of torque balance [19]. To solve the problems that affect a doctor’s true perception of the feedback force information, a gravity compensation model, friction compensation model, and inertial force compensation model is established [20] and a force compensation control strategy is adopted. For this compensation method, it is necessary to establish an accurate dynamic model to achieve better results.

Some ideas from the above algorithm can be applied to the gravity compensation, but most of them only collect space-limited points for gravity compensation. The compensation accuracy is relatively poor, and the operating efficiency is very low, so it is not suitable for industrial sites that require high precision and high beat force control. At the same time, there are relatively few studies considering the inertial force compensation algorithm, because most six-dimensional force sensors do not have inertial force sensing devices (such as multi-axis acceleration sensors) to sense inertia force. This paper uses a six-dimensional force sensor that integrates gravity/inertial force compensation, and is inspired by the dynamic parameter identification method based on the excitation trajectory [21]. A fast and efficient algorithm for gravity compensation and inertial force compensation is proposed, and it can meet the requirements of high-speed and high-tempo operations in industrial sites.

## 2. Gravity Compensation Algorithm

### 2.1. Analysis of the Influence of Load Gravity

**Figure 1:**
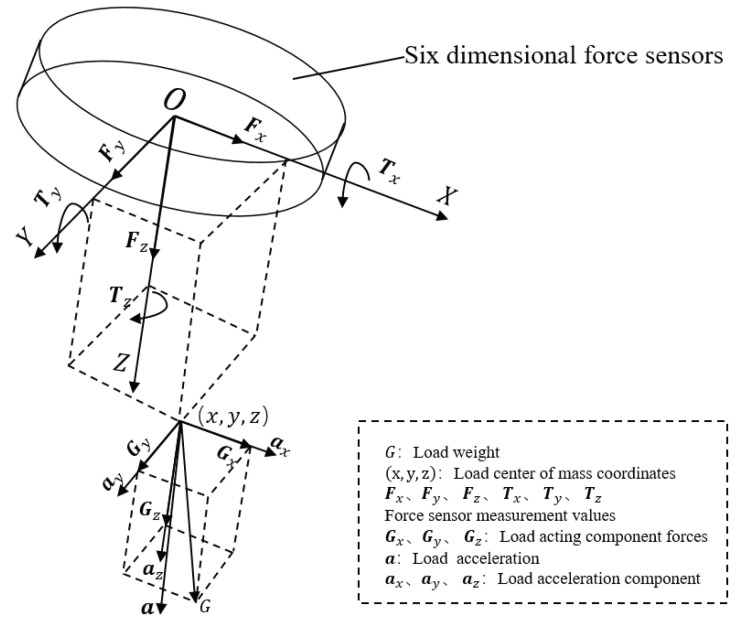
Diagram of gravity compensation.

In this paper, a force sensor is installed at the end of the robot to sense the contact forces with the external environment. In general, a six-dimensional force sensor is selected to measure the three-dimensional orthogonal forces $(F_{x},F_{y},F_{z})$ and three-dimensional orthogonal moment $(T_{x},T_{y},T_{z})$ in the arbitrary force systems in space, as shown in Figure 1. In the static case, the force and moment data perceived by the end of the robot include four parts: (1) sensor system error; (2) load gravity; (3) load inertia force; (4) external contact force on the load.

The six-dimensional force sensor is generally installed between the flange and the end tool. When the load posture changes with the operation, although the direction of gravity is always vertically down, the component of the load gravity on each axis of the sensor coordinate system is different. The coordinate system of the six-dimensional force sensor is set to FT, the robot end coordinate system is *E*, the robot base coordinate system is *B*, the world coordinate system is *W*, and the homogeneous transformation matrix of the sensor coordinate system with relative to the robot end coordinate system is ${}_{\mathrm{FT}}^{E}T$. For any given end position ${}_{E}^{B}T$, the gravity of the end tool has components on each axis in the FT coordinate system. If the z-axis of the sensor is consistent with the direction of gravity, the end gravity acts only on the z-axis of the *FT* coordinate system. The purpose of gravity compensation is to eliminate the gravity influence of the end tool, and the gravity and center of gravity parameters of the end load can be obtained through the gravity compensation algorithm.

### 2.2. Force and Torque of Six-Dimensional Force Sensor

In general, the six-dimensional force sensor has an initial zero value due to errors such as processing and calibration, the zero value of the three force components are recorded as $F_{x_{0}}$, $F_{y_{0}}$, and $F_{z_{0}}$, and the zero value of the three torque components are recorded as $T_{x_{0}}$, $T_{y_{0}}$, and $T_{z_{0}}$. The origin of the sensor coordinate system is ***O***, and the direction of the coordinate system is consistent with that given by the sensor manufacturer. Assuming that the load gravity is ***G*** and the coordinate of the load center of gravity in the sensor coordinate system is (x,y,z), then the force components of the load gravity in the sensor coordinate system are $G_{x}$, $G_{y}$, and $G_{z}$, and the force moments are $T_{gx}$, $T_{gy}$ and $T_{gz}$. The formula shown in Equation (1) can be obtained from the relationship between force and torque by the right-hand rule.

(1) $$\left\{\begin{array}{c} T_{gx}=G_{y}\times z-G_{z}\times y\\ T_{gy}=G_{z}\times x-G_{x}\times z\\ T_{gz}=G_{x}\times y-G_{y}\times x \end{array}\right.$$

The force component feedback values measured by the six-dimensional force sensor are recorded as $F_{x}$, $F_{y}$, and $F_{z}$, respectively, and the torque component feedback values are recorded as $T_{x}$, $T_{y}$, and $T_{z}$, respectively. When there is no external force at the end of the robot, the measured value of the sensor, the force component/moment of the load gravity under the sensor, and the zero value of the sensor are shown in Equation (2).

(2) $$\left\{\begin{array}{c} G_{x}=F_{x}-F_{x_{0}}\\ G_{y}=F_{y}-F_{y_{0}}\\ G_{z}=F_{z}-F_{z_{0}}\\ T_{gx}=T_{x}-T_{x_{0}}\\ T_{gy}=T_{y}-T_{y_{0}}\\ T_{gz}=T_{z}-T_{z_{0}} \end{array}\right.$$

Substituting Equation (2) into Equation (1) yields

(3) $$\left\{\begin{array}{c} T_{x}=F_{y}\times z-F_{z}\times y+T_{x_{0}}+F_{z_{0}}\times y-F_{y_{0}}\times z\\ T_{y}=F_{z}\times x-F_{x}\times z+T_{y_{0}}+F_{x_{0}}\times z-F_{z_{0}}\times x\\ T_{z}=F_{x}\times y-F_{y}\times x+T_{z_{0}}+F_{y_{0}}\times x-F_{x_{0}}\times y \end{array}\right.$$

In Equation (3), $F_{x_{0}}$, $F_{y_{0}}$, $F_{z_{0}}$, $T_{x_{0}}$, $T_{y_{0}}$, $T_{z_{0}}$, and x,y,z are all constants, so that:

(4) $$\left\{\begin{array}{c} k_{1}=T_{x_{0}}+F_{z_{0}}\times y-F_{y_{0}}\times z\\ k_{2}=T_{y_{0}}+F_{x_{0}}\times z-F_{z_{0}}\times x\\ k_{3}=T_{z_{0}}+F_{y_{0}}\times x-F_{x_{0}}\times y \end{array}\right.$$

### 2.3. Load Center of Gravity Coordinate Calculation

To obtain the load center coordinates, Equation (4) can be taken into Equation (3), and Equation (5) can be obtained:

(5) $$\left[\begin{array}{c} T_{x}\\ T_{y}\\ T_{z} \end{array}\right]=\left[\begin{array}{cccccc} 0 & -F_{z} & F_{y} & 1 & 0 & 0\\ F_{z} & 0 & -F_{x} & 0 & 1 & 0\\ -F_{y} & F_{x} & 0 & 0 & 0 & 1 \end{array}\right]\left[\begin{array}{c} x\\ y\\ z\\ k_{1}\\ k_{2}\\ k_{3} \end{array}\right]$$

In order to obtain accurate identification results, N sets of data can be collected (N≥3), and the normal vectors at the end of the robot in at least three postures are not coplanar, that is linearly independent. Then Equation (5) can be reconstructed as Equation (6):

(6) $$\left[\begin{array}{c} T_{x_{1}}\\ T_{y_{1}}\\ T_{z_{1}}\\ T_{x_{2}}\\ T_{y_{2}}\\ T_{z_{2}}\\ ...\\ T_{x_{N}}\\ T_{y_{N}}\\ T_{z_{N}} \end{array}\right]=\left[\begin{array}{cccccc} 0 & -F_{z_{1}} & F_{y_{1}} & 1 & 0 & 0\\ F_{z_{1}} & 0 & -F_{x_{1}} & 0 & 1 & 0\\ -F_{y_{1}} & F_{x_{1}} & 0 & 0 & 0 & 1\\ 0 & -F_{z_{2}} & F_{y_{2}} & 1 & 0 & 0\\ F_{z_{2}} & 0 & -F_{x_{2}} & 0 & 1 & 0\\ -F_{y_{2}} & F_{x_{2}} & 0 & 0 & 0 & 1\\ ...\\ 0 & -F_{z_{N}} & F_{y_{N}} & 1 & 0 & 0\\ F_{z_{N}} & 0 & -F_{x_{N}} & 0 & 1 & 0\\ -F_{y_{N}} & F_{x_{N}} & 0 & 0 & 0 & 1 \end{array}\right]\left[\begin{array}{c} x\\ y\\ z\\ k_{1}\\ k_{2}\\ k_{3} \end{array}\right]$$

Convert Equation (6) to the form of linear equation, as shown in Equation (7):

(7) τ=F·p

of which,

(8) $$p={\left[\begin{array}{cccccc} x & y & z & k_{1} & k_{2} & k_{3} \end{array}\right]}^{T}$$

After a finite number of elementary linear changes, the matrix ***F*** can be verified to be a non-full rank matrix with rank(F)=3N, so that the Equation (7) is actually overdetermined equation, so the corresponding least squares solution is:

(9) $$p={\left(F^{T}F\right)}^{-1}\cdot F^{T}\tau$$

the coordinate values (x,y,z) and the constant $k_{1},k_{1},k_{3}$ of gravity in the coordinate system of the six-dimensional force sensor can be solved from the above formulas.

### 2.4. Calculation of Base Mounting Inclination, Sensor Zero Point, and Load Gravity

In the actual process of industrial robot installation, there is often a flatness error between the robot base and the ground, that is, the z-axis of the robot coordinate system is not necessarily parallel to the gravity direction, so there is an installation inclination angle.

The installation inclinations angle U and V between the robot base and the world coordinate system are the deviation angles between the machine base and the world coordinate system in the x-axis and y-axis directions, respectively, which satisfies Equation (10).

(10) $${}_{B}^{W}R=\left[\begin{array}{ccc} 1 & 0 & 0\\ 0 & \cos U & -\sin U\\ 0 & \sin U & \cos U \end{array}\right]\left[\begin{array}{ccc} \cos V & 0 & \sin V\\ 0 & 1 & 0\\ -\sin V & 0 & \cos V \end{array}\right]$$

Equation (11) is the rotation matrix of the sensor coordinate system relative to the base coordinate system, where the angles of rotation around the *Z, Y, X* axes in the base coordinate system are represented by *A, B, C*, respectively, which can be obtained from the robot control system. It is assumed that the robot tool coordinate system coincides with the sensor coordinate system.

The direction vector of gravity in the world coordinate system is expressed by Equation (11).

(11) $$\begin{array}{cc} {}_{FT}^{B}R & =R_{Z}\left(A\right)R_{Y}\left(B\right)R_{X}\left(C\right)\\ & =\left[\begin{array}{ccc} \cos A & -\sin A & 0\\ \sin A & \cos A & 0\\ 0 & 0 & 1 \end{array}\right]\left[\begin{array}{ccc} \cos B & 0 & \sin B\\ 0 & 1 & 0\\ -\sin B & 0 & \cos B \end{array}\right]\left[\begin{array}{ccc} 1 & 0 & 0\\ 0 & \cos C & -\sin C\\ 0 & \sin C & \cos C \end{array}\right] \end{array}$$

(12) $$g_{W}=\left[\begin{array}{c} 0\\ 0\\ -1 \end{array}\right]$$

According to the above equation, the directional vector of the load in the sensor coordinate system can be obtained as Equation (13).

(13) $$g_{FT}={}_{B}^{FT}R\cdot {}_{W}^{B}R\cdot g_{W}={}_{FT}^{B}R^{T}\cdot {}_{B}^{W}R^{T}\cdot g_{W}={}_{FT}^{B}R^{T}\left[\begin{array}{c} \cos U\cdot \sin V\\ -\sin U\\ -\cos U\cdot \cos V \end{array}\right]$$

Suppose the gravity of the load is G, and Equation (14) can be obtained from Equation (2).

(14) $$\left[\begin{array}{c} F_{x}\\ F_{y}\\ F_{z} \end{array}\right]=\left[\begin{array}{c} G_{x}\\ G_{y}\\ G_{z} \end{array}\right]+\left[\begin{array}{c} F_{x_{0}}\\ F_{y_{0}}\\ F_{z_{0}} \end{array}\right]={}_{FT}^{B}R^{T}\left[\begin{array}{c} G\cdot \cos U\cdot \sin V\\ -G\cdot \sin U\\ -G\cdot \cos U\cdot \cos V \end{array}\right]+\left[\begin{array}{c} F_{x_{0}}\\ F_{y_{0}}\\ F_{z_{0}} \end{array}\right]$$

Let the constant term be:

(15) $$\left\{\begin{array}{c} L_{x}=G\cdot cosU\cdot sinV\\ L_{y}=-G\cdot sinV\\ L_{z}=-G\cdot cosU\cdot cosV \end{array}\right.$$

Equation (14) can be arranged as follows:

(16) $$\left[\begin{array}{c} F_{x}\\ F_{y}\\ F_{z} \end{array}\right]=\left[\begin{array}{cc} {}_{FT}^{B}R^{T} & I \end{array}\right]\left[\begin{array}{c} L_{x}\\ L_{y}\\ L_{z}\\ F_{x_{0}}\\ F_{y_{0}}\\ F_{z_{0}} \end{array}\right]$$

where I is the unit matrix of 3 × 3. In order to ensure the accuracy of the calculation, the *A*, *B*, *C* values of *N* groups of robots in different postures are collected, and the ${}_{FT}^{B}R^{T}$ matrix of *N* gestures is obtained, and the following equation is obtained.

(17) $$\left[\begin{array}{c} F_{x_{1}}\\ F_{y_{1}}\\ F_{z_{1}}\\ F_{x_{2}}\\ F_{y_{2}}\\ F_{z_{2}}\\ ...\\ F_{x_{N}}\\ F_{y_{N}}\\ F_{z_{N}} \end{array}\right]=\left[\begin{array}{cc} {}_{FT}^{B}R_{1}^{T} & I\\ {}_{FT}^{B}R_{2}^{T} & I\\ ... & ...\\ {}_{FT}^{B}R_{N}^{T} & I \end{array}\right]\left[\begin{array}{c} L_{x}\\ L_{y}\\ L_{z}\\ F_{x_{0}}\\ F_{y_{0}}\\ F_{z_{0}} \end{array}\right]$$

The above equation can be transformed into a system of linear equation:

(18) f=Rl

of which:

(19) $$l={\left[\begin{array}{cccccc} L_{x} & L_{y} & L_{z} & F_{x_{0}} & F_{y_{0}} & F_{z_{0}} \end{array}\right]}^{T}$$

The least squares solution of Equation (18) is:

(20) $$l={\left(R^{T}R\right)}^{-1}\cdot R^{T}f$$

The zero points $F_{x_{0}}$, $F_{y_{0}}$, $F_{z_{0}}$ and the constants $L_{x}$,$L_{y}$,$L_{z}$ of the six-dimensional force transducer can be solved from Equation (20). the zero point of the moment of the sensor can be solved from Equation (4):

(21) $$\left\{\begin{array}{c} T_{x_{0}}=k_{1}-F_{z_{0}}\times y+F_{y_{0}}\times z\\ T_{y_{0}}=k_{2}-F_{x_{0}}\times z+F_{z_{0}}\times x\\ T_{z_{0}}=k_{3}-F_{y_{0}}\times x+F_{x_{0}}\times y \end{array}\right.$$

The gravity of the load and the mounting inclination of the robot can be obtained from Equation (15):

(22) $$G=\sqrt{{L_{x}}^{2}+{L_{y}}^{2}+{L_{z}}^{2}}$$

(23) $$\left\{\begin{array}{c} U=\arcsin(-\frac{L_{y}}{G})\\ V=\arctan(-\frac{L_{x}}{L_{z}}) \end{array}\right.$$

### 2.5. Calculation of External Force Perception

The three components of the load gravity in the coordinate system of the six-dimensional force sensor can be obtained from Equations (13) and (15):

(24) $$\left[\begin{array}{c} G_{x}\\ G_{y}\\ G_{z} \end{array}\right]=G\cdot g_{FT}={}_{FT}^{B}R^{T}\left[\begin{array}{c} L_{x}\\ L_{y}\\ L_{z} \end{array}\right]$$

Substituting Equation (24) into Equation (1), the moment components $T_{gx}$, $T_{gy}$, $T_{gz}$ of gravity acting on the sensor coordinate system can be obtained.

The value of the sensor feedback contains the zero value, the force/moment component caused by the load gravity and the external force, so the external force/moment value can be calculated as follows:

(25) $$\left\{\begin{array}{c} F_{ex}=F_{x}-F_{x_{0}}-G_{x}\\ F_{ey}=F_{y}-F_{y_{0}}-G_{y}\\ F_{ez}=F_{z}-F_{z_{0}}-G_{z}\\ T_{ex}=T_{x}-T_{x_{0}}-T_{gx}\\ T_{ey}=T_{y}-T_{y_{0}}-T_{gy}\\ T_{ez}=T_{z}-T_{z_{0}}-T_{gz} \end{array}\right.$$

## 3. Inertial Force Compensation Algorithm

### 3.1. Analysis of the Influence of Load Inertia Force

When the manipulator performs the operation with a certain acceleration, the inertia mass effect will be produced by the end tool due to the acceleration produced by the load end of the sensor (such as fixture, actuator, etc.). The inertial force F′ and inertial moment T′ will be generated by the end load under the influence of acceleration, and the force and moment perceived by the sensor will be affected by these two values; therefore, the inertial force and inertial moment need to be compensated to obtain more accurate measurements values, which provides the correct force feedback values for the force control algorithm.

### 3.2. Inertial Force Models and Compensation Algorithms

In the process of fast motion od the robot, due to the action of load, the inertial force $F_{inert}$ and inertial moment $T_{inert}$ will be produced at the end of the robot in the variable speed motion, and the following equations are obtained from Newton’s second law and the law of rigid body rotation:

(26) $$F_{inert}=m\cdot a$$

(27) $$T_{inert}=J\cdot \alpha$$

where *m* is the mass of the load, a is the acceleration of the load movement, J is the rotational inertia of the load, and α is the angular acceleration of the load rotation. The acceleration and angular acceleration can be read directly by the accelerometer installed in the six-dimensional force sensor, the load mass is identified by gravity compensation, and the rotational inertia is calculated according to theoretical mechanics.

The measured value of the sensor $\left(F_{meas}\;and\;T_{meas}\right)$ is composed of two components: the desired tracking force/moment $\left(F_{des}\;and\;T_{des}\right)$ and the inertia force/moment value.

(28) $$\left\{\begin{array}{c} F_{meas}=F_{des}+F_{inert}\\ T_{meas}=T_{des}+T_{inert} \end{array}\right.$$

The inertia forces/moment is compensated by an equivalent compensation force and moment:

(29) $$\left\{\begin{array}{c} F_{meas}=F_{des}+F_{inert}+F_{comp}\\ T_{meas}=T_{des}+T_{inert}+T_{comp} \end{array}\right.$$

where the compensating force/moment is defined as follows, and then the compensation of inertia forces/moments is realized.

(30) $$\left\{\begin{array}{c} F_{comp}=-F_{inert}\\ T_{comp}=-T_{inert} \end{array}\right.$$

## 4. Fast Gravity/Inertial Force Identification Method Based on Excitation Trajectories

### 4.1. The Combined Forces of Gravity and Inertia Are Expressed

Based on the contents in Section 2 and Section 3, the gravity and inertial force can be calculated, respectively, but it takes a long time to identify. In order to realize the rapid identification of gravity and inertial forces, a fast identification method based on excitation trajectories will be introduced in this section.

Firstly, the expression method and the solution equation of the resultant force of gravity and inertia force are introduced. With the combination Equations (14), (15) and (28), the comprehensive expressions of gravity and inertia force can be obtained:

(31) $$\left[\begin{array}{c} F_{x}\\ F_{y}\\ F_{z} \end{array}\right]={}_{FT}^{R}R_{T}\left[\begin{array}{c} G\cdot \cos U\cdot \sin V\\ -G\cdot \sin U\\ -G\cdot \cos U\cdot \cos V \end{array}\right]+\left[\begin{array}{c} F_{x_{0}}\\ F_{y_{0}}\\ F_{z_{0}} \end{array}\right]+\frac{G}{g}\left[\begin{array}{c} a_{x}\\ a_{y}\\ a_{z} \end{array}\right]$$

The above Equation (31) can be sorted into the form of Equation (32), and the comprehensive Equation (33) of gravity moment and inertia moment can be arranged.

Let $H_{x}=F_{y_{0}}z-F_{z_{0}}y+T_{x_{0}}$, $H_{y}=F_{z_{0}}x-F_{x_{0}}z+T_{y_{0}}$ and $H_{z}=F_{x_{0}}y-F_{y_{0}}x+T_{z_{0}}$. Then, Equation (33) can be sorted into Equation (34).

For Equations (32) and (34), sensor data are usually collected from *N* sets of robots at different attitudes to obtain Equation (35) shown below.

(32) $$\left[\begin{array}{c} F_{x}\\ F_{y}\\ F_{z} \end{array}\right]=\left[\begin{array}{cc} {}_{FT}^{R}R^{T} & I \end{array}\right]\left[\begin{array}{c} L_{x}\\ L_{y}\\ L_{z}\\ F_{x_{0}}+\frac{G}{g}a_{x}\\ F_{y_{0}}+\frac{G}{g}a_{y}\\ F_{z_{0}}+\frac{G}{g}a_{z} \end{array}\right]$$

(33) $$\left\{\begin{array}{c} T_{x}=F_{z}y-F_{y}z+F_{x_{0}}z-F_{z_{0}}y+T_{x_{0}}+\frac{G}{g}\left(a_{z}y-a_{y}z\right)\\ T_{y}=F_{x}z-F_{z}x+F_{z_{0}}x-F_{x_{0}}z+T_{y_{0}}+\frac{G}{g}\left(a_{x}z-a_{z}x\right)\\ T_{z}=F_{y}x-F_{x}y+F_{x_{0}}y-F_{y_{0}}x+T_{z_{0}}+\frac{G}{g}\left(a_{y}x-a_{x}y\right) \end{array}\right.$$

(34) $$\left[\begin{array}{c} T_{x}\\ T_{y}\\ T_{z} \end{array}\right]=\left[\begin{array}{cccccc} 0 & F_{z}+\frac{G}{g}a_{y} & -F_{y}-\frac{G}{g}a_{y} & 1 & 0 & 1\\ -F_{z}-\frac{G}{g}a_{z} & 0 & F_{x}+\frac{G}{g}a_{x} & 0 & 1 & 0\\ F_{y}+\frac{G}{g}a_{y} & -F_{x}-\frac{G}{g}a_{x} & 0 & 0 & 0 & 1 \end{array}\right]\left[\begin{array}{c} x\\ y\\ z\\ H_{x}\\ H_{y}\\ H_{z} \end{array}\right]$$

(35) $$\left[\begin{array}{c} F_{x_{1}}\\ F_{y_{1}}\\ F_{z_{1}}\\ F_{x_{2}}\\ F_{y_{2}}\\ F_{z_{2}}\\ ...\\ F_{x_{N}}\\ F_{y_{N}}\\ F_{z_{N}} \end{array}\right]=\left[\begin{array}{cc} {}_{FT}^{B}{R_{1}}^{T} & I\\ {}_{FT}^{B}{R_{2}}^{T} & I\\ ... & ...\\ {}_{FT}^{B}{R_{N}}^{T} & I \end{array}\right]\left[\begin{array}{c} L_{x}\\ L_{y}\\ L_{z}\\ F_{x_{0}}+\frac{G}{g}a_{x}\\ F_{y_{0}}+\frac{G}{g}a_{y}\\ F_{z_{0}}+\frac{G}{g}a_{z} \end{array}\right]$$

The above Equation (35) is transformed into a system of linear equations:

(36) F=RL

of which,

(37) $$L={\left[\begin{array}{cccccc} L_{x} & L_{y} & L_{z} & F_{x_{0}}+\frac{G}{g}a_{x} & F_{y_{0}}+\frac{G}{g}a_{y} & F_{z_{0}}+\frac{G}{g}a_{z} \end{array}\right]}^{T}$$

The corresponding least squares solutions to Equation (37) are Equations (38) and (39).

(38) $$L={\left(R^{T}R\right)}^{-1}\cdot R^{T}F$$

(39) $$\left[\begin{array}{c} T_{x_{1}}\\ T_{y_{1}}\\ T_{z_{1}}\\ T_{x_{2}}\\ T_{y_{2}}\\ T_{z_{2}}\\ ...\\ T_{x_{N}}\\ T_{y_{N}}\\ T_{z_{N}} \end{array}\right]=\left[\begin{array}{cccccc} 0 & F_{z_{1}}+\frac{G}{g}a_{z_{1}} & -F_{y_{1}}-\frac{G}{g}a_{y_{1}} & 1 & 0 & 0\\ -F_{z_{1}}-\frac{G}{g}a_{z_{1}} & 0 & F_{x_{1}}+\frac{G}{g}a_{x_{1}} & 0 & 1 & 0\\ F_{y_{1}}+\frac{G}{g}a_{y_{1}} & -F_{x_{1}}-\frac{G}{g}a_{x_{1}} & 0 & 0 & 0 & 1\\ 0 & F_{z_{2}}+\frac{G}{g}a_{z_{2}} & -F_{y_{2}}-\frac{G}{g}a_{y_{2}} & 1 & 0 & 0\\ -F_{z_{2}}-\frac{G}{g}a_{z_{2}} & 0 & F_{x_{2}}+\frac{G}{g}a_{x_{2}} & 0 & 1 & 0\\ F_{y_{2}}+\frac{G}{g}a_{y_{2}} & -F_{x_{2}}-\frac{G}{g}a_{x_{2}} & 0 & 0 & 0 & 1\\ ... & ... & ... & ... & ... & ...\\ 0 & F_{z_{N}}+\frac{G}{g}a_{z_{N}} & -F_{y_{N}}-\frac{G}{g}a_{y_{N}} & 1 & 0 & 0\\ -F_{z_{N}}-\frac{G}{g}a_{z_{N}} & 0 & F_{x_{N}}+\frac{G}{g}a_{x_{N}} & 0 & 1 & 0\\ F_{y_{N}}+\frac{G}{g}a_{y_{N}} & -F_{x_{N}}-\frac{G}{g}a_{x_{N}} & 0 & 0 & 0 & 1 \end{array}\right]\left[\begin{array}{c} x\\ y\\ z\\ H_{x}\\ H_{y}\\ H_{z} \end{array}\right]$$

Equation (39) is transformed into a system linear equation:

(40) T=RH

of which,

(41) $$H={\left[\begin{array}{cccccc} x & y & z & H_{x} & H_{y} & H_{z} \end{array}\right]}^{T}$$

The corresponding least squares solution to Equation (41) is:

(42) $$H={\left(R^{T}R\right)}^{-1}\cdot R^{T}T$$

In Equations (38) and (42), the unknown parameters are the load gravity *G*, inclination angles of base installation *U*, *V*, force values of sensor zero point $F_{x_{0}}$, $F_{y_{0}}$, $F_{z_{0}}$, tool center of gravity coordinates *x*, *y*, *z*, and $T_{x_{0}}$, $T_{y_{0}}$, $T_{z_{0}}$, a total of 12 parameters, *N* (N≥12) groups of sensor data under different attitudes are collected, and 12 unknown parameters can be obtained by substitution into Equations (38) and (42).

### 4.2. Excitation Trajectory Design

Each of the unknown parameters of the gravity/inertia force can be identified by changing the attitude of the end (that is, related to the 4–6 axes). In order to make the selected attitude represent the identification space as much as possible, an optimal joint reference trajectory needs to be designed. In order to excite the relevant parameters of the end 4–6 axis joint, the equations related to the joint parameters are found, that is, the moment equations under inertia force are considered as excitation equations, such as Equation (43).

(43) $$\left[\begin{array}{c} T_{a_{x}}\\ T_{a_{y}}\\ T_{a_{z}} \end{array}\right]=\left[\begin{array}{ccc} 0 & a_{z} & -a_{y}\\ -a_{z} & 0 & a_{x}\\ a_{y} & -a_{x} & 0 \end{array}\right]\left[\begin{array}{c} \frac{G}{g}x\\ \frac{G}{g}y\\ \frac{G}{g}z \end{array}\right]$$

The acceleration of the load is the vector sum of the acceleration generated by the rotating of the load around the end coordinate system and the acceleration of the coordinate system itself, namely:

(44) $$\left[\begin{array}{c} a_{x}\\ a_{y}\\ a_{z} \end{array}\right]=\left[\begin{array}{c} x\\ y\\ z \end{array}\right]\times \alpha +\left[\begin{array}{c} a_{x_{0}}\\ a_{y_{0}}\\ a_{z_{0}} \end{array}\right]$$

where α is the angular acceleration of the robot end coordinate system and ${\left[\begin{array}{ccc} a_{x_{0}} & a_{y_{0}} & a_{z_{0}} \end{array}\right]}^{T}$ is the acceleration of the end coordinate system itself, Equation (45) can be obtained by substituting the above Equation (43).

(45) $$\left[\begin{array}{c} T_{a_{x}}\\ T_{a_{y}}\\ T_{a_{z}} \end{array}\right]=\left[\begin{array}{ccc} 0 & x\alpha_{y}-y\alpha_{x}+a_{z_{0}} & -z\alpha_{x}+x\alpha_{z}-a_{y_{0}}\\ -x\alpha_{y}+y\alpha_{x}-a_{z_{0}} & 0 & y\alpha_{z}-z\alpha_{y}+a_{x_{0}}\\ z\alpha_{x}-x\alpha_{z}+a_{y_{0}} & -y\alpha_{+}z\alpha_{y}-a_{x_{0}} & 0 \end{array}\right]\left[\begin{array}{c} \frac{G}{g}x\\ \frac{G}{g}y\\ \frac{G}{g}z \end{array}\right]$$

The above equation can be expressed as follows:

(46) $$T_{a}=a\cdot p$$

where the acceleration a is a function of $\theta_{4}$, $\theta_{5}$ and $\theta_{6}$, which will be denoted as a=f(q4,q5,q6). p is the part except a in the above formula. The objective function of the optimization is denoted as minf(a), the physical meaning of this is to fill the whole space with the end of the robot as much as possible. The excitation trajectory of each joint is expressed by a Fourier series of finite terms, then the angular displacement $q_{i}$, angular velocity ${\dot{q}}_{i}$, and angular acceleration ${\ddot{q}}_{i}$ of joint *i* of the robot can be expressed as:

(47) $$q_{i}\left(t\right)=\sum_{l=1}^{N}\left[\frac{a_{i,j}}{w_{f}l}\sin\left(w_{f}lt\right)-\frac{b_{i,j}}{w_{f}l}\cos\left(w_{f}lt\right)\right]$$

(48) $${\dot{q}}_{i}\left(t\right)=\sum_{l=1}^{N}\left[a_{i,j}\cos\left(w_{f}lt\right)+b_{i,j}\sin\left(w_{f}lt\right)\right]$$

(49) $${\ddot{q}}_{i}\left(t\right)=\sum_{l=1}^{N}w_{f}l\left[-a_{i,j}\sin\left(w_{f}lt\right)+b_{i,j}\cos\left(w_{f}lt\right)\right]$$

where *N* represents the number of terms of sine and cosine, $w_{f}=2\pi f_{f}$ is the fundamental angular frequency, and $a_{i,l}$ and $b_{i,l}$ are the parameters to be determined. Since gravity compensation only involves the 4th, 5th, and 6th axes at the end of the robot arm, a total of 6N parameters need to be optimized, and the optimization objective is recorded as:

(50) minf(a)

To meet the constraint conditions of robot joints, the following constraint expressions are given:

(51) $$\left|q_{i}\left(t\right)\right|\leq \left|\sum_{l=1}^{N}\frac{1}{w_{f}l}\sqrt{{a_{i,l}}^{2}+{b_{i,l}}^{2}}\right|\leq q_{i,\max}$$

(52) $$\left|{\dot{q}}_{i}\left(t\right)\right|\leq \left|\sum_{l=1}^{N}\sqrt{{a_{i,l}}^{2}+{b_{i,l}}^{2}}\right|\leq {\dot{q}}_{i,\max}$$

(53) $$\left|{\ddot{q}}_{i}\left(t\right)\right|\leq \left|\sum_{l=1}^{N}w_{f}l\sqrt{{a_{i,l}}^{2}+{b_{i,l}}^{2}}\right|\leq {\ddot{q}}_{i,\max}$$

The start and stop constraint equations are as follow:

(54) $$q_{i}\left(t_{0}\right)=\sum_{l=1}^{N}\left(-\frac{b_{i,l}}{w_{f}l}\right)=0$$

(55) $${\dot{q}}_{i}\left(t_{0}\right)=\sum_{l=1}^{N}a_{i,l}=0$$

(56) $${\ddot{q}}_{i}\left(t_{0}\right)=\sum_{l=1}^{N}w_{f}lb_{i,l}=0$$

The factors such as the “unrepresentative” attitude of the identification point and the floating of sensor data are considered, and $M_{a}$, a, and p are all disturbed, and the equations after the disturbance are as follows:

(57) $$T_{a}+\delta T_{a}=\left(a+\delta a\right)\cdot \left(p+\delta p\right)$$

Subtracting from the original equation gives:

(58) $$\delta T_{a}=a\cdot \delta p+\delta a\cdot p+\delta a\cdot \delta p$$

(59) $$\delta p=a^{-}\left(\delta T_{a}-\delta a\cdot p-\delta a\cdot \delta p\right)$$

Therefore,

(60) $$∥\delta p∥\leq ∥a^{-}∥\left(∥\delta T_{a}∥+∥\delta a∥\cdot ∥p∥+∥\delta a∥\cdot ∥\delta p∥\right)$$

can be solved for:

(61) $$∥\delta p∥\leq \frac{∥a^{-}∥}{1-∥a^{-}∥∥\delta a∥}\left(∥\delta T_{a}∥+∥\delta a∥\cdot ∥p∥\right)$$

Since $T_{a}=a\cdot p$, then $∥T_{a}∥\leq ∥a∥\cdot ∥p∥$

Thus, Equation (59) can be rewritten as:

(62) $$∥\delta p∥\leq \frac{∥a^{-}∥}{1-∥a^{-}∥∥\delta a∥}\left(\frac{∥\delta T_{a}∥}{∥T_{a}∥}\cdot ∥a∥\cdot ∥p∥+∥\delta a∥\cdot ∥p∥\right)$$

Then the relative error of the identification parameter b is:

(63) $$\frac{∥\delta p∥}{∥p∥}\leq \frac{∥a^{-}∥\cdot ∥a∥}{1-∥a^{-}∥∥\delta a∥}\left(\frac{∥\delta T_{a}∥}{∥T_{a}∥}+\frac{∥\delta a∥}{∥a∥}\right)$$

Since $\frac{∥\delta T_{a}∥}{∥T_{a}∥}+\frac{∥\delta a∥}{∥a∥}$ is a relative perturbation term with a very small value, the error in the identification parameter matrix p is mainly caused by $∥a^{-}∥\cdot ∥a∥$. The condition number is introduced here as an optimization objective function for the excitation trajectory, that is:

(64) $$Cond\left(a\right)=∥a^{-}∥\cdot ∥a∥$$

The relative error of parameter *b* can be obtained by substituting this equation into parameter *b*:

(65) $$\frac{∥\delta p∥}{∥p∥}\leq \frac{Cond(a)}{1-Cond\left(a\right)\frac{∥\delta a∥}{∥a∥}}\left(\frac{∥\delta T_{a}∥}{∥T_{a}∥}+\frac{∥\delta a∥}{∥a∥}\right)$$

Optimized objective function:

(66) $$\min f\left(a\right)=∥a^{-}∥∥a∥$$

Under the conditions of the satisfying the position, velocity, and acceleration of each joint of the robot, when the value of the optimization objective function minf(a) is at its minimum, it means that the external disturbance of the external disturbance to the identification parameter matrix p is the least, and the corresponding parameters $a_{i,j}$ and $b_{i,j}$ are the corresponding parameters of the excitation trajectory, the purpose of the excitation trajectory is to make the posture of the end of the robot diverse, so it can be assumed that the position vector of the center of mass of the workpiece in the coordinate system of the robot end is a known quantity p, and it can be substituted into Equation (45) to excite the representative posture of the robot.

### 4.3. Excitation Trajectory Curve

**Figure 2:**
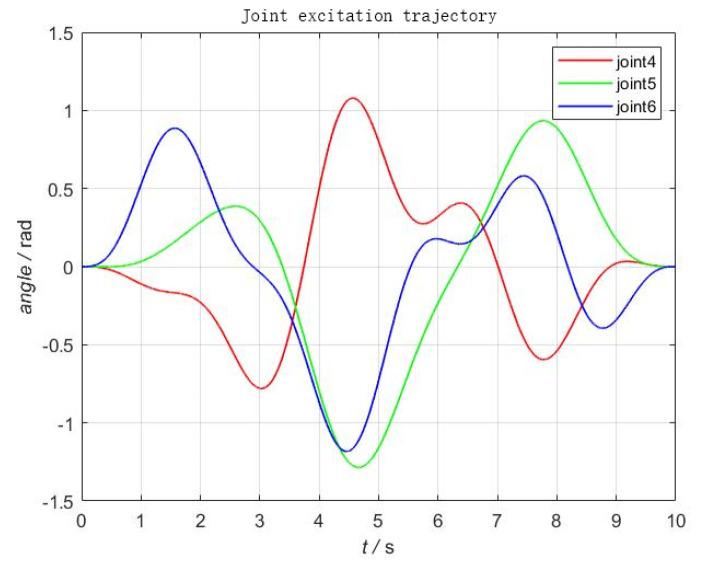
Joint-time trajectory.

**Figure 3:**
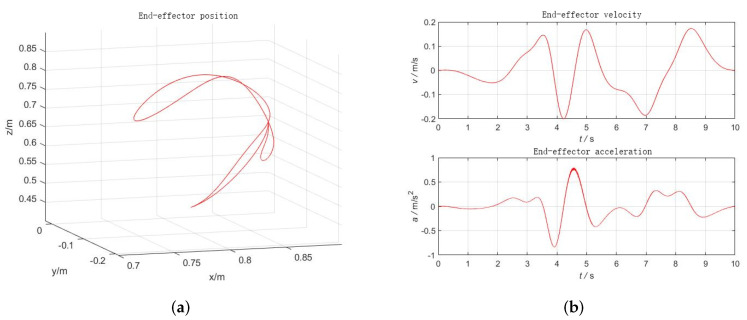
End-effector position, velocity, and acceleration profiles. (**a**) Position; (**b**) Velocity, acceleration.

The number of sine and cosine terms in the Fourier series is taken as N=5, the fundamental frequency $f_{f}$ is selected as 0.1 Hz, and the sampling frequency is taken as 100 Hz, a total of 1000 samples are taken to calculate the matrix a. To achieve the excitation of each joint parameter, the fmincon function in Matlab is selected and the excitation trajectory can be obtained by inputting the optimization objective function and each constraint into this function. The angular variations of joints 4–6 are shown in Figure 2, and the corresponding Cartesian space end position, velocity, and acceleration curves are shown in Figure 3.

According to the above excitation trajectory, the sensor data under various attitudes are obtained, and the 12 parameters, such as the load gravity *G*, the installation inclination angle of machine *U*, *V*, the zero point force values of the sensor $F_{x_{0}}$, $F_{y_{0}}$, $F_{z_{0}}$, the tool center of gravity coordinates *x*, *y*, *z*, and $T_{x_{0}}$, $T_{y_{0}}$, $T_{z_{0}}$ can be identified by Equations (38) and (42).

## 5. Experimental Verification and Results

### 5.1. Experimental Platform Introduction and Verification

**Figure 4:**
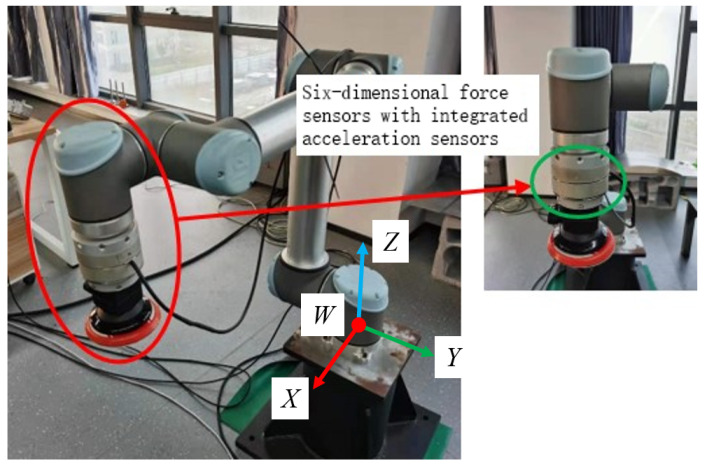
Physical test scene diagram.

The UR10 robot system and the self-developed six-dimensional force sensor with acceleration sensing were used as the platform for this experiment (the sensor performance test can be found in [22], also shown in Table 1). The self-developed six-dimensional force sensor can directly measure angle, angular velocity, and angular acceleration. These data can be directly used for gravity and inertial compensation. A test load was mounted on the end of the robot; the experimental scene diagram is shown in Figure 4. According to the design scheme of the excitation trajectory in Section 4.2, an identification trajectory with a movement time of 10 s was designed, as shown in Figure 2 and Figure 3. In order to realize the synchronous periodic motion control of the robot and the synchronous acquisition of the sensors, an industrial computer platform with the Linux Real-Time operating system was built. The 8 ms communication control between the industrial computer and the UR10 can be realized by using the servoj instructions provided by the UR10. According to the designed identification trajectory, a total of 1250 discrete points of motion were interpolated, and the data of 1250 groups of sensors were synchronously collected. The data set can be substituted into Equation (42) to obtain the identification results. The identification results include load mass and center of gravity position, the initial force/torque value, and the installation inclination angle of the foundation; the results are shown in Table 2.

### 5.2. Experimental Results and Analysis

**Figure 5:**
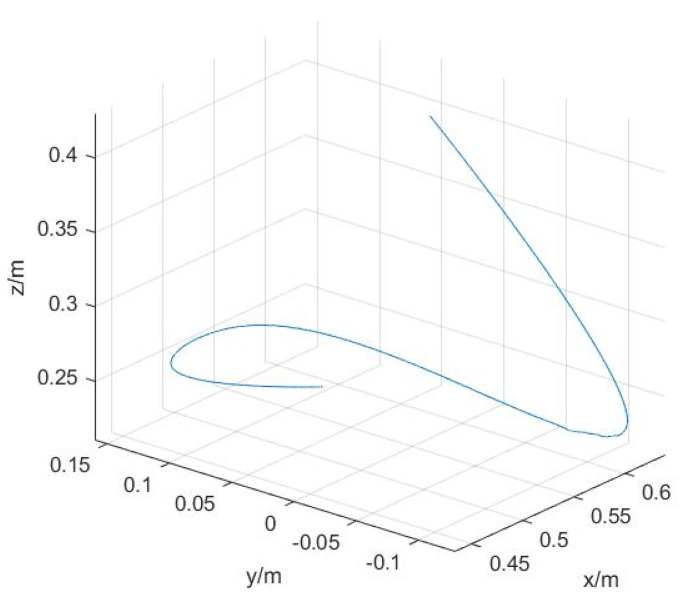
Test trajectories in Cartesian space.

**Figure 6:**
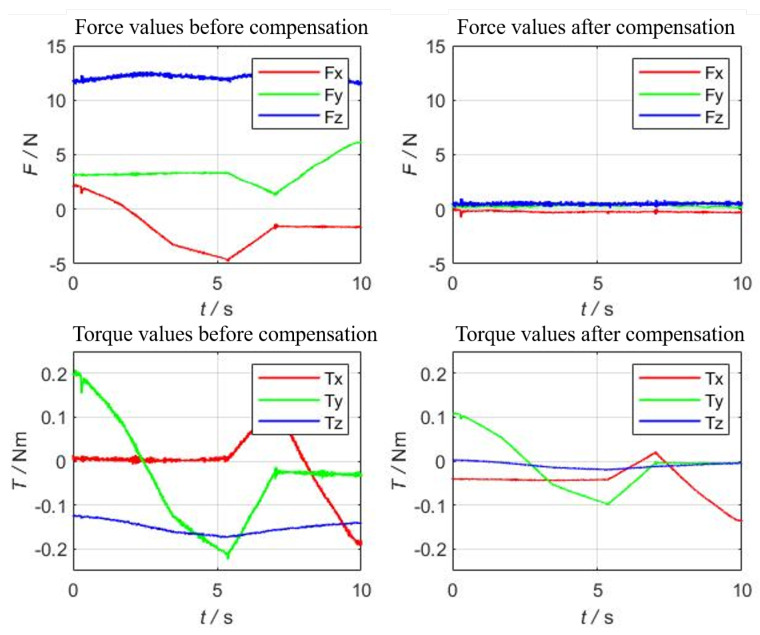
Results after compensation of gravitational and inertial forces.

In order to verify whether the identification result is accurate, multiple trajectories with acceleration in Cartesian space is further designed to verify the compensation result, including straight lines, arcs, variable posture curves, and arbitrary drag trajectory. One representative curve was chosen to show the compensation results as shown in Figure 5, that is a arbitrary curve with varying poses. The robot’s motion data and sensor values are collected synchronously, and the corresponding results after gravity and inertial force compensation can be obtained after entering from Equations (28)–(30), several sets of experiments were performed, and one set of results is shown in Figure 6. The other compensation results are consistent with this set of results; the result shows the compensation effect remain in the same values. It can be seen from Figure 6 that before compensation, the force value of the z-axis exceeds 10 N, and the torque value exceeds 0.5 Nm. The compensated force value is within 0.5 N, and the torque value is within 0.2 Nm, which meets the needs of industrial field applications.

**Figure 7:**
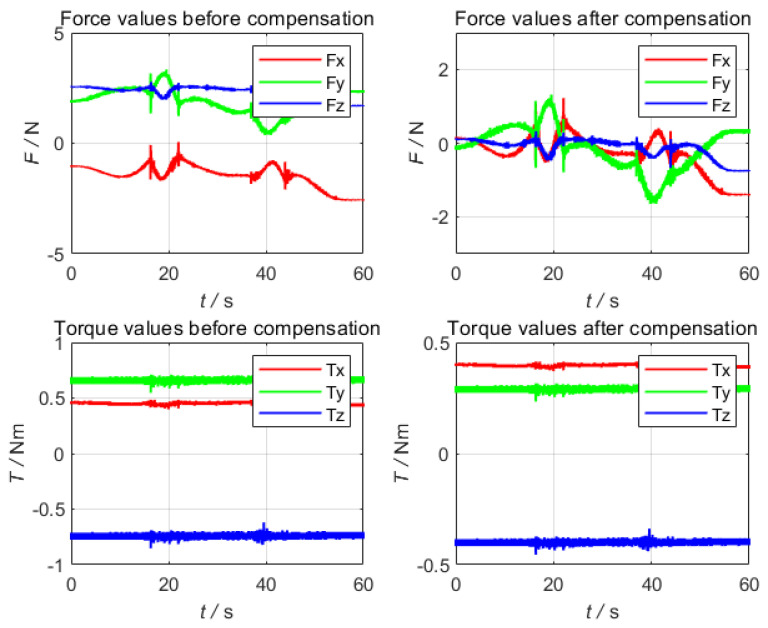
The result of the traditional compensation algorithm.

Compared with other algorithms, one of the advantages of this algorithm is that the identification time is short. The identification time of the traditional six-point method or the least square method with limited points is more than 60 s, and the result of the traditional compensation algorithm as shown in Figure 7. It can be seen that the compensation result exceeds 1 N in the force direction and 0.4 Nm in the torque direction.

The existing algorithm is only used for gravity compensation, not for inertial force compensation, so it is impossible to design an identification trajectory that is too fast. The algorithm proposed in this paper can satisfy both low-speed and high-speed conditions. It not only compensates for gravity, but also compensates for high-speed or heavy-load conditions. The identification of payload can be completed only through the designed excitation trajectory. Due to the rapid identification, the algorithm can be applied to the occasions that require high time tempo in the industrial field.

## 6. Conclusions

For the precise force control of the robot, gravity, and inertia, force compensation is particularly critical, and the effect of force control is directly affected by its identification and compensation accuracy. In this paper, the effects of inertia force caused by high speeds or large loads is considered, by using the self-developed multi-dimensional force sensor with acceleration sensor, the inertia force, and inertia moment of the robot are measured directly, and the measurement data are provided directly for inertia force identification. A fast identification method based on the excitation trajectory is proposed to quickly and accurately identify the relevant parameters of the robot tool under fast/large loads, which can shorten the identification time to 10 s and the efficiency of identification is greatly improved. Through the verification of the test trajectory, it is found that the accuracy of force and moment compensation can be improved to less than 0.5 N and 0.2 Nm, respectively. The problem of the traditional algorithm is that the identification time is long, and only the gravity compensation is considered, which is not suitable for high speed or heavy load. The algorithm mentioned in this paper is very helpful for industrial applications, not only can shorten the identification time, but also can compensate for high-speed or large-load motion at the same time. Through this algorithm, the sensor can accurately sense the external force, laying the foundation for the next force control application.

## Figures and Tables

**Table 1:** The characteristic information of the sensor.

Tpye	GTL96003AXX (Self-Developed)
Range	Fx/Fy: 300 N, Fz: 500 N, Tx/Ty/Tz: 25 Nm
Weight	700 g
Size	Ø80 mm × 40 mm
Protection level	IP65
Overload capacity	500% FS
Resolution	0.1 N/0.02 Nm
Acceleration sensing accuracy	acceleration ≤ 0.01 g, angular velocity ≤ 0.05 deg/s

**Table 2:** Identification result.

$F_{x_{0}}$ (N)	$F_{y_{0}}$ (N)	$F_{z_{0}}$ (N)	$T_{x_{0}}$ (Nm)	$T_{y_{0}}$ (Nm)	$T_{z_{0}}$ (Nm)
−0.6672	0.8565	0.3538	0.0228	0.0084	0.0080
*x* (cm)	*y* (cm)	*z* (cm)	*G* (N)	*U* (${}^{\circ }$)	*V* (${}^{\circ }$)
0.5	0.2	5.1	8.862	−9.8716	−5.3709

## Data Availability

Not Applicable.
